# Suitability and Sufficiency of Telehealth Clinician-Observed, Participant-Collected Samples for SARS-CoV-2 Testing: The iCollect Cohort Pilot Study

**DOI:** 10.2196/19731

**Published:** 2020-06-25

**Authors:** Jodie L Guest, Patrick S Sullivan, Mariah Valentine-Graves, Rachel Valencia, Elizabeth Adam, Nicole Luisi, Mariko Nakano, Jeannette Guarner, Carlos del Rio, Charles Sailey, Zoe Goedecke, Aaron J Siegler, Travis H Sanchez

**Affiliations:** 1 Rollins School of Public Health Emory University Atlanta, GA United States; 2 Molecular Testing Labs Vancouver, WA United States; 3 School of Medicine Emory University Atlanta, GA United States

**Keywords:** COVID-19, testing, home testing, telehealth, pilot study, diagnostic, diagnosis

## Abstract

**Background:**

The severe acute respiratory coronavirus 2 (SARS-CoV-2) pandemic calls for expanded opportunities for testing, including novel testing strategies such as home-collected specimens.

**Objective:**

We aimed to understand whether oropharyngeal swab (OPS), saliva, and dried blood spot (DBS) specimens collected by participants at home and mailed to a laboratory were sufficient for use in diagnostic and serology tests of SARS-CoV-2.

**Methods:**

Eligible participants consented online and were mailed a participant-collection kit to support collection of three specimens for SARS-CoV-2 testing: saliva, OPS, and DBS. Participants performed the specimen collection procedures during a telehealth video appointment while clinical observers watched and documented the suitability of the collection. The biological sufficiency of the specimens for detection of SARS-CoV-2 by reverse transcriptase–polymerase chain reaction and serology testing was assessed by laboratorians using visual inspection and quantification of the nucleic acid contents of the samples by ribonuclease P (RNase P) measurements.

**Results:**

Of the enrolled participants,153/159 (96.2%) returned their kits, which were included in this analysis. All these participants attended their video appointments. Clinical observers assessed that of the samples collected, 147/153 (96.1%) of the saliva samples, 146/151 (96.7%) of the oropharyngeal samples, and 135/145 (93.1%) of the DBS samples were of sufficient quality for submission for laboratory testing; 100% of the OPS samples and 98% of the saliva samples had cycle threshold values for RNase P <30, indicating that the samples contained sufficient nucleic acid for RNA-PCR testing for SARS-CoV-2.

**Conclusions:**

These pilot data indicate that most participant-collected OPS, saliva, and DBS specimens are suitable and sufficient for testing for SARS-CoV-2 RNA and serology. Clinical observers rated the collection of specimens as suitable for testing, and visual and quantitative laboratory assessment indicated that the specimens were biologically sufficient. These data support the utility of participant-collected and mailed-in specimens for SARS-CoV-2 testing.

**International Registered Report Identifier (IRRID):**

RR2-10.2196/19054

## Introduction

The United States is experiencing expansive spread of severe acute respiratory syndrome coronavirus 2 (SARS-CoV-2) as part of a global pandemic of the virus [1]. The rapid rise in the number of cases of infection in the United States has taxed multiple aspects of our health care systems, including capacity for testing for the virus and supply chains for personal protective equipment (PPE), specimen collection swabs, and supplies and equipment for people requiring hospital care. There is a national call to expand opportunities for testing for SARS-CoV-2, to reduce the need for PPE and specimen collection swabs currently required for testing of SARS-CoV-2, and to test for SARS-CoV-2 outside of health care facilities [2-4].

Decisions about coronavirus disease (COVID-19) mitigation policies must be informed by the best epidemiologic information, which requires rapid scaleup of SARS-CoV-2 testing. Currently, testing is limited, and many people with clinical indications cannot receive a test [5]. For instance, as of April 8, 2020, the US rate of SARS-CoV-2 testing was 7131 tests per 1 million people, or 2,360,512 overall since January 10, 2020 [6]. Testing has mainly focused on those most severely ill and requiring hospitalization; this low testing rate and targeted testing provides undercounted and biased estimates that do not inform an understanding of the epidemiology of SARS-CoV-2 infection or enable optimal recommendation of control measures [7]. South Korea currently has the highest rate of testing in the world; this has likely contributed to their successful mitigation of their COVID-19 disease epidemic [6,8]. Based on data from the COVID Tracking Project, at least 1 million US residents should be tested every week (0.3% of the population) during this phase of the pandemic [6,9].

We must find scalable and acceptable ways of reaching more people with testing without overburdening our already taxed health care systems. Novel testing strategies such as rapid diagnostic tests, serological tests, and participant-collected specimens could improve our ability to screen large numbers of people quickly and provide new understanding of the extent of exposure, disease, and recovery without compounding the need for health care personnel and PPE to collect the specimens. The US Food and Drug Administration (FDA) has approved self-collection of midturbinate swabs and anterior nares swabs for reverse transcriptase–polymerase chain reaction (RT-PCR) testing under the supervision of a health care provider in health care settings [10]; however, as of April 11, 2020, there are no FDA-approved options for unsupervised participant collection of specimens for SARS-CoV-2 RT-PCR or testing for antibodies to SARS-CoV-2. These options would be important in the response to the epidemic because they would provide efficient methods to conduct large-scale epidemiologic studies, provide options for testing people without causing crowding in provider offices, and enable testing without requiring the use of the scarce PPE required for providers administering in-person tests.

Commercial HIV test kits using self-collection of specimens have been on the market in the United States since 1996. Concerns were reported for these tests regarding self-collection of samples for HIV testing, including having to wait for results, potential mixup of mailed specimens, and cost [11,12]. However, the benefit assessment for the kit showed that these concerns were offset by the convenience and privacy of specimen collection at home and strong public interest [11-16]. The FDA has approved tests of home-collected specimens for a wide variety of analytes and infectious diseases, including HIV, hepatitis C, and sexually transmitted infections. These are typically marketed through a company that provides a clinician who orders the test, discusses the results with the patient if needed, and assumes regulatory responsibility for infectious disease reporting requirements.

A primary concern with at-home tests is the ability of users to correctly conduct the tests. Several studies have examined how well untrained users can conduct HIV self-tests with oral fluid or whole blood fingersticks; most of these studies concluded that participants were able to conduct the tests successfully [17-22]. The Pre-Exposure Prophylaxis at Home (PrEP@Home) system was developed to allow people to mail in home-collected specimens and to provide the remote laboratory testing needed for HIV PrEP use while removing the substantial burden of in-person laboratory visits [7]. Based on the high acceptability of and preference for PrEP@Home specimen collection relative to laboratory collection, we anticipate that home sample collection kits for SARS-CoV-2 would be well utilized despite requiring participant collection of multiple specimens at multiple sites.

Given the ongoing pace of SARS-CoV-2 transmission with inadequate testing, the iCollect study aimed to understand the viability of home collection of specimens as a pathway to increase SARS-CoV-2 testing for people who may not otherwise require immediate medical attention, who may need to obtain follow-up testing while they are convalescent, or who may be assessed as part of an epidemiological study.

We observed and evaluated the ability of a convenience sample of adults in the continental United States to collect a dried blood spot (DBS) card specimen, a saliva tube specimen, and an oropharyngeal swab (OPS) specimen at home that were all suitable and sufficient for laboratory testing for SARS-CoV-2 RNA and serology. DBS specimens have been used for other infectious disease serology tests [23]. Saliva specimens are a plausible specimen type for SARS-CoV-2 testing because salivary glands have been described as a possible reservoir for viral persistence [24] and viral shedding in saliva or sputum can persist for weeks after infection [25]. Saliva may also have diagnostic utility because it can be a vehicle for oral mucosal cells [26]. The FDA has currently issued emergency use authorization (EUA) approvals for two saliva tests, although both tests involve saliva or oral fluid collection by a health care provider [27,28].

To assess the suitability of the specimens, the specimen collection was observed through a telehealth session with clinician observers, including physicians, nurses, and MD candidates working under the supervision of a physician. To assess biological sufficiency, laboratorians evaluated the specimens through laboratory accession screening and RNA-PCR testing. We report the suitability (by clinician observation) and sufficiency (by laboratory assessment of specimens) of the participant-collected samples to be analyzed for SARS-CoV-2 RNA and serology.

## Methods

### Participants, Setting, and Eligibility

The methods for the study have been previously described [29]. Briefly, participants were eligible if they were ≥18 years of age, resided in the United States, had never been diagnosed with a bleeding disorder, were able to read and understand English without assistance, were willing to provide valid contact information so that study testing kits could be mailed to participants, had access to a mobile phone, tablet, or computer with a camera, and were willing to be observed by a clinician while completing the specimen collection processes.

### Recruitment

Participants were recruited through two methods. First, we offered enrollment to people who had participated in a previous research study of willingness to self-test for SARS-CoV-2 infection and who agreed to be contacted for participation in future research studies [30]. Second, we shared a link with information about the study within networks of people symptomatic for COVID-19 or at risk for SARS-CoV-2 infection, including through networks of first responders. Participants who accessed the link to the information about the study were offered the opportunity to consent to online screening. Those who consented were screened for eligibility, and those who were eligible were provided with informed consent documents and a contact telephone number and email address to ask questions about the study. Participants were offered US $50 for completion of all study activities (eg, baseline survey, observed participant-collection session, return of specimens by mail, and post-collection survey).

### Data Sources and Collection

#### Participant-Collection Specimen Kit

All participants were mailed a study participant-collection specimen kit composed of a cardboard mailing box, instruction sheets for self-collection of specimens (available in [29]), a saliva collection tube, a specimen collection swab, a vial of viral transport medium, a self-retracting lancet, an alcohol pad, a Whatman dried blood spot collection card, a gauze pad, a small self-adhesive bandage, a biohazard bag, and a prepaid return mailing label.

#### Clinician-Observed Participant Collection Video Appointment

Participants were sent a link by email to schedule their specimen collection video appointment using a Health Insurance Portability and Accountability Act (HIPPA)-compliant videoconference service. During the video/specimen collection appointment, the clinical observers did not instruct the participants, instead directing them to perform the specimen collection procedures using the instruction sheets [29] in the test kit as if they had been provided the kit and instructions without external observation. The clinical observers documented their observations while the participant collected the specimens and recorded their determination of whether the collection appeared to be suitable for submission for laboratory testing and clinical decision making. Clinical observers were instructed not to respond to questions about how to collect the samples but to redirect participants to the written instructions provided. Clinical observers were instructed to intervene only if the participant was performing an action that might pose a risk to themselves. Study case report forms provided space for the clinician to document whether questions were asked during the collection and the provider’s observations about the collection [29].

In addition to the provider’s overall assessment of the suitability of the specimen, three specimen-specific checklists of items were used by the clinician to document adherence to directions (eg, whether each step in the instructions was followed and completed by the participant; see Multimedia Appendix 1 in [29]). After completing the at-home collection, participants were asked to package the specimens and mail the completed specimens directly to the central study laboratory using the provided mailer.

### Laboratory Assessment of Biological Sufficiency

The main outcome of interest was the biological sufficiency of the specimens for testing by RT-PCR and for detection of antibodies by serology testing. The biological sufficiency of the OPS specimens for PCR was assessed by evaluating the total nucleic acid in the specimen using ribonuclease P (RNase P) measurements as previously described [31]. Briefly, saliva and OPS specimens were subjected to nucleic acid extraction using the Thermo Kingfisher platform (Thermo Fisher Scientific). Extracts were tested for human RNase P by RT-PCR with the Thermo SARS CoV-2 testing kit v1. We considered saliva and OPS with cycle threshold (C_t_) values <30 to contain sufficient collections of nucleic acid (as a proxy for collection of biological material) [29]. We compared the C_t_ values of the participant-collected and shipped saliva and OPS specimens to a laboratory reference set of 100 saliva specimens and 100 clinician-collected OPS specimens that were transported directly to the laboratory on ice after collection from a separate clinical population, and we described the differences in the median C_t_ value between the clinician-collected specimens and the clinician-observed, participant-collected specimens. To assess the biological sufficiency of the DBS cards, we performed a three-point quality check on the cards, assessing the visual appearance of the blood spot, whether the blood had soaked through the paper, and whether the circles were filled, according to our previously reported method for other DBS specimens [29].

### Ethical Approval

Approval for this study was obtained from the Institutional Review Board at Emory University, and the specifics of the protocol have been previously published [29].

## Results

### Participants

We enrolled 159 participants in the iCollect cohort pilot study; 61 (38.4%) were male, 91 (57.2%) were female, 1 (0.6%) was genderqueer, and 1 (0.6%) was multiple gender (Table 1). Most were non-Hispanic white/Caucasian (110/159, 69.2%) and were less than 40 years of age (99/159, 62.3%); 13/159 (8.2%) were 60 years or older. The 159 participants reported residence in the US regions of South/Southeast (57, 35.8%), Northeast (43, 28.3%), Midwest (27, 17.0%), West (14, 15.1%), and Northwest (8, 5.0%). Most reported at least one symptom of COVID-19 at the time of the survey: 51 of the 159 enrolled participants (32.1%) reported no symptoms, 56 (35.2%) had 1-3 relevant symptoms, 29 (17.6%) had 4-5 symptoms, and 9 (5.7%) reported 6-8 of the listed symptoms (Table 1).

**Figure 1:**
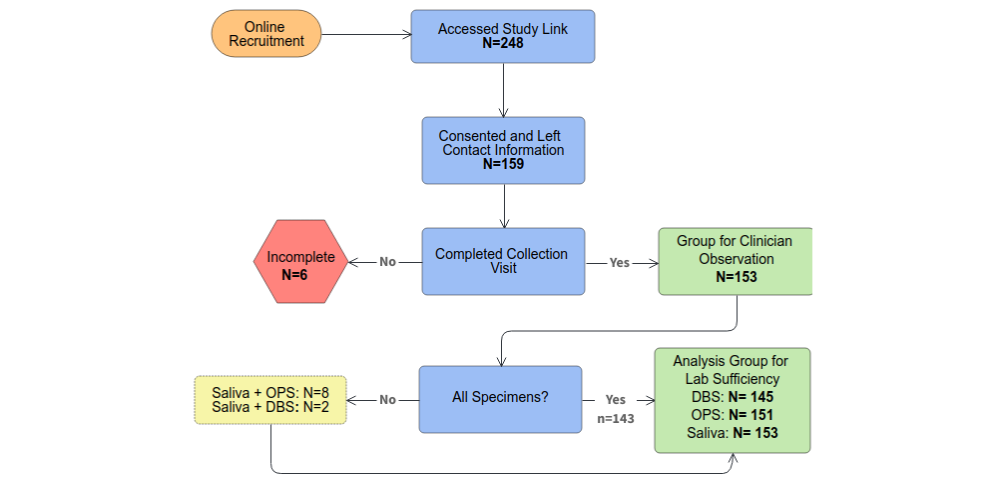
Participant and specimen flow of the iCollect study. DBS: dried blood spot. OPS: oropharyngeal swab.

A total of 228 respondents accessed the registration link. A total of 159 participants were eligible (Figure 1), gave consent, and provided contact information for the kit mailing. We mailed kits to 159 participants; 153 (96.2%) of these participants scheduled a video appointment, and all 153 (100.0%) completed that appointment. The mean time for all video appointments was 32 minutes (median 29 minutes, range 13-143 minutes). Of the 153 participants who attended a video appointment, 143 (93%) completed collections of all three samples (Figure 1). DBS was the most commonly uncollected sample; however, only 8 participants did not collect a DBS card. Thus, the analytic sample for clinician assessment of suitability and the laboratory assessment of sufficiency was 153 saliva specimens, 151 OPSs, and 145 DBS cards (Figure 1).

**Table 1:** Characteristics of the iCollect pilot study participants (N=159).

Characteristic		n (%)
**Age (years)**		
	18-21	7 (4.4)
	22-29	56 (35.2)
	30-39	36 (22.6)
	40-49	23 (14.5)
	50-59	24 (15.1)
	60-69	8 (5.0)
	≥70	5 (3.1)
**Race/ethnicity**		
	White/Caucasian, non-Hispanic	110 (69.2)
	Black/African American, non-Hispanic	12 (7.5)
	Latino/Hispanic	22 (13.8)
	Asian/Pacific Islander, non-Hispanic	8 (5.0)
	Native American/Alaska native	0 (0.0)
	Mixed race, non-Hispanic	2 (1.2)
	Not reported	5 (3.1)
**Current gender**		
	Female	91 (57.2)
	Male	61 (38.4)
	Genderqueer	1 (0.6)
	Multiple	1 (0.6)
	Not reported	5 (3.1)
**Residence (US region)**		
	Northeast	43 (28.3)
	Midwest	27 (17.0)
	South/Southeast	57 (35.8)
	Northwest	8 (5.0)
	West	24 (15.1)
**Symptoms**		
	Shortness of breath	24 (15.1)
	Fever	9 (5.7)
	Cough	58 (36.5)
	Sneezing	36 (22.6)
	Sore throat	34 (21.4)
	Headache	49 (30.8)
	Diarrhea	18 (11.3)
	Myalgia	15 (9.4)
	Feeling of being unwell	41 (25.8)
	None	51 (32.1)
	Not reported	5 (3.1)
**Number of symptoms**		
	0	51 (32.1)
	1	21 (13.2)
	2	20 (12.6)
	3	15 (9.4)
	4	22 (13.8)
	5	6 (3.8)
	6	4 (2.5)
	7	4 (2.5)
	8	1 (0.6)
	Not reported	15 (9.4)

### Clinical Observer Assessment of Suitability for Laboratory Testing

Clinical observers assessed that 147/153 (96%) of the saliva samples, 146/151 (96.7%) of the oropharyngeal samples, and 135/145 (93.1%) of the DBS samples were of sufficient quality to be submitted for laboratory testing (Tables 2 and 3). Clinician reasons for lack of suitability are also reported in Tables 2 and 3.

**Table 2:** Numbers of samples collected in the iCollect study with clinician assessment of the suitability of the collection procedures (n=153).

Sample	Total attempted collections observed, n (%)	Total samples collected, n (%)
DBS^a^	148^b^ (96.7)	145^c^ (94.8)
Saliva	153 (100.0)	153 (100.0)
OPS^d^	152^e^ (99.3)	151^f^, 98.7

^a^DBS: dried blood spot.

^b^Five DBS collections were not observed: 2 participants did not see the instructions, 2 did not have the instructions, and 1 experienced anxiety/fainting when drawing blood and did not complete the process.

^c^Three DBS samples were not collected: no blood.

^d^OPS: oropharyngeal swab.

^e^One OPS collection was not observed: no swab in the kit.

^f^One OPS sample was not collected: the participant vomited while attempting to collect it.

**Table 3:** Numbers of samples assessed as suitable and unsuitable for laboratory testing.

Sample	Clinician assessed as suitable, n (%)	Clinician assessed as unsuitable, n (%)
DBS^a^ (n=145)	135 (93.1)	10^b^ (6.9)
Saliva (n=153)	147 (96.1)	6^c^ (3.9)
OPS^d^ (n=151)	146 (96.7)	5^e^ (3.3)

^a^DBS: dried blood spot.

^b^Ten DBS samples were unsuitable: 3 had <3 spots, 3 participants pressed their finger into the paper, 2 participants did not fill the spots completely, 1 unknown, and 1 participant did not wash their hands.

^c^Six saliva samples were insufficient: 4 did not invert the tube, 1 did not use the instructions and missed steps, 1 contained lots of foam.

^d^OPS: oropharyngeal swab.

^e^Five oropharyngeal swabs were insufficient: 3 participant did not swab long enough (<20 seconds), 1 participant only held the swab against the roof of the mouth, and 1 participant swabbed their cheeks.

Clinical observers also documented compliance with specific steps in the instructions for each specimen type; these data are presented in Tables 4-6. For DBS collection, the most common errors were touching the specimen collection paper when making the spots (29/148,19.6%) and not completely filling all the circles (52/148, 35.1%). The median number of filled spots was 5; 3 filled spots are required for standard serology assessments in our laboratory, and 137/148 (92.6%) of participants filled at least 3 spots.

**Table 4:** Clinician-documented participant actions when collecting DBS samples and conducting COVID-19 self-testing during the iCollect study (n=148).

Participant action		n (%)
Labeled DBS^a^ card, including name, date of birth, and date of collection		145^b^ (98.0)
Did not touch blood collection paper		136^c^ (91.9)
Washed hands before collection		143 (96.6)
Cleaned finger with alcohol pad		143 (96.6)
Used lancet on side of finger		133 (89.9)
Did not touch paper while making spots		119^b^ (80.4)
**Filled spots completely**		
	All spots	96 (64.9)
	Some spots	38 (25.7)
	No spots	14 (9.5)
Set the card aside to dry		142^d^ (95.9)
**Number of spots filled**		
	0	4 (2.7)
	1	2 (1.3)
	2	5 (3.4)
	3	9 (6.1)
	4	15 (10.1)
	5	113 (76.4)

^a^DBS: dried blood spot.

^b^One assessment missing.

^c^Four assessments missing.

^d^Three assessments missing.

**Table 5:** Clinician-documented participant actions when collecting saliva samples and conducting COVID-19 self-testing during the iCollect study (n=153).

Participant action	n (%)
Did not drink, eat, or smoke immediately before or during collection	152^a^ (99.3)
Washed hands before collection	135 (88.2)
Rinsed their mouth with water before collection	112^a^ (73.2)
Placed their lips over the funnel when providing the saliva sample	145^a^ (94.8)
Filled the tube to the red indicator line	146^b^ (95.4)
Unscrewed the funnel and put on the cap	152^a^ (99.3)
Inverted the vial 20 times	134^a^ (87.6)
Removed the barcode label and applied it to the tube	145^a^ (94.8)
Wrote their date of birth on the barcode label	140^b^ (91.5)
Placed the specimen in the biohazard bag and sealed the bag	150^b^ (98.0)

^a^One assessment missing.

^b^Two assessments missing.

**Table 6:** Clinician-documented participant actions when collecting oropharyngeal swabs and conducting COVID-19 self-testing in the iCollect study (n=152).

Participant action	n (%)
Did not drink, eat, or smoke immediately before or during collection	151^a^ (99.3)
Washed hands before collection	135 (88.8)
Did not let the swab touch anything before or after collection	148^a^ (97.4)
Inserted the swab in their mouth and swabbed each side for approximately 20 seconds	137^a^ (90.1)
Placed the swab in the collection tube	150^a^ (98.7)
Broke the swab at the score line	151^a^ (99.3)
Placed the lid on the collection tube and tightened it	150^a^ (98.7)
Wrote their date of birth on the tube	141^b^ (92.8)
Placed the specimen in the biohazard bag and sealed the bag	150^a^ (98.7)

^a^One assessment missing.

^b^Two assessments missing.

### Laboratory Staff Assessment of Biological Sufficiency for Biological Testing 

Data are presented for the first 101 OPSs, first 123 saliva specimens, and first 137 DBS cards processed by the laboratory. For the saliva specimens, all specimens except three had C_t_ values for RNase P <30 (the value of one specimen was 30.6; 98% of specimens met our pre-specified threshold [29] for sufficient nucleic acid for detection of target RNA). The median C_t_ for the saliva specimens was 19.5 (IQR 18.8-20.8). For oropharyngeal swabs, all specimens had C_t_ values for RNase P <30, meeting our pre-specified threshold [29] for sufficient nucleic acid for detection of target RNA. The median C_t_ for the OPS specimens was 23.9 (IQR 21.0-25.3). We compared the median C_t_ for OPS patient-collected specimens under clinician observation to the C_t_ values of 18 clinician-collected OPS swabs processed in the same central laboratory by the Wilcoxon rank sum test. The results indicated that there was no significant difference in the C_t_ (and, by inference, no difference in the concentrations of nucleic acid in the specimens) between participant-collected and clinician-collected OPS (median self-collected 23.9; median provider-collected 23.7; *P*=.70). For the 140 DBS cards evaluated, the median number of usable 6 millimeter punches was 3 (IQR 1-5). In terms of saturation, 70/140 (50.0%) were classified as good, 31/140 (22.1%) were classified as fair, and 38/140 (27.1%) were classified as poor; 1 card (0.7%) was assessed as having no blood. In terms of dryness (1=wet, 10=dry), the median dryness was 10 (IQR 9-10). The minimum dryness was 4.

## Discussion

### Principal Findings

US and global response to the SARS-CoV-2 pandemic desperately requires at-home sample collection both to detect people who are infected with the SARS-CoV-2 virus and for the measurement and monitoring of antibody response to the infection. Unlike nasopharyngeal swab collection, OPS, saliva, and DBS collections do not require any medical training. The level of testing that has been performed to date in the United States is limited for multiple reasons; important solutions are to diversify the types of specimens that have sufficient biological material to be accurately evaluated for SARS-CoV-2 infection (as assessed by RNase P) and immune response (as assessed by saturation and number of usable blood spots) and to diversify the locations in which these specimens can be collected. This study aimed to provide evidence of whether specimens collected at home for SARS-CoV-2 diagnosis are suitable (as assessed by clinical observers) and are sufficient (as assessed by laboratorians). Our results indicate that the collection of the specimens by the participants at home for the diagnosis of SARS-CoV-2 infection and serologic response was suitable as judged by clinical observers. Additionally, the OPS and saliva specimens were judged by objective measures to be sufficient for analysis of SARS-CoV-2 PCR by laboratorians. Most DBS cards contained sufficient samples for testing; however, the laboratorian-rated quality of saturation was variable. Our assessment did not validate these specimens as appropriate specimen types for use for SARS-CoV-2 testing; however, we did assess that the samples had adequate biological material to support testing. Both OPS and saliva have been determined by the FDA to be suitable specimen types for SARS-CoV-2 detection assays [32,33]. Specimens were tested for SARS-CoV-2 RNA by RT-PCR (saliva, oropharyngeal swab) and for IgG and IgM antibodies (dried blood spot, saliva) and IgA antibody (saliva); however, the results are not reported here because the primary intent of this analysis was to describe whether home-collected specimens were suitable and sufficient for RT-PCR and serology testing.

A major finding of our study was that home collection of specimens returned by mail is highly acceptable as a means of submitting specimens for testing for SARS-CoV-2; 143/153 (93.5%) of the participants who were sent kits completed collection of all the specimens and returned the kits. These data confirm findings from a separate study assessing the willingness of people to collect and return specimens for SARS-CoV-2–related testing [30]. In that study, participants were very willing to submit saliva and oropharyngeal swab specimens but were slightly less likely to report willingness to submit dried blood spot specimens. Our study suggests that the extent to which participants actually collect specimens is consistent with previous reports of their willingness to do so, as reported by different participants in an online survey (eg, the saliva collection was the most complete, and participants in a separate study reported being most willing to provide saliva specimens) [30] These data are also consistent with the acceptability of at-home specimen collection for other health conditions, including a long history of the use of at-home dried blood spot collection for HIV diagnosis [34,35]. In our prior work, we found that video instructions may be helpful in increasing the successful collection of specimens, including DBS specimens. We will consider evaluating video instructions as a complement to printed instructions, and we will continue to evaluate the quality of the collected specimens, especially the saturation and completed number of dried blood spot specimens.

To our knowledge, our study is unique in that we used both telehealth to provide clinician observation of participant-collected specimens and rigorous laboratory assessment to determine the sufficiency of those same specimens. We intend that these data will help create a bridge between current regulatory approvals for self-collection of specimens for SARS-CoV-2 diagnosis in clinical settings (eg, OPS; and participant-collected anterior nares swabs, participant-collected OPS, and participant-collected saliva when those specimens are collected under the supervision of a health care provider) and eventual regulatory review of at-home self-collection specimens for laboratory testing. We believe that this study addresses one important component that would support a transition from clinician-observed collection of these specimens to fully unobserved self-collection of specimens that are returned by mail: that the quality of the specimens for diagnostic purposes must be equivalent to clinician-collected specimens. Our evidence in this regard is strong because we incorporated both the professional opinions of clinical observers and objective assessment of the sufficiency of the samples by laboratorians.

However, we recognize that ultimate implementation of at-home self-collection of specimens for diagnosis of SARS-CoV-2 will also be dependent on other important factors. First, it is important that the materials that are sent out in at-home kits are safe, including consideration of the safety of the components of those kits even if they are not used as directed in the test kit instructions. We believe that this can be addressed by review of the material safety data sheets for the components of the kits and by considering modifications of the kits (eg, providing viral transport media in child-resistant tubes) to further improve the safety of the kit components in diverse household settings. Second, stability tests will be required to indicate whether the diagnostic sufficiency of specimens is compromised by conventional shipping processes, delayed shipping, or shipping in extreme environmental conditions. There are well-characterized protocols for such stability studies [36]; ensuring that the test performs as expected under a variety of environmental conditions and after shipping delays is an important part of assuring the diagnostic integrity of the task and, ultimately, the overall performance of the testing approach.

It is also important to view the consideration of deploying at-home participant-collection specimen kits through a broader lens to examine the potential risks and benefits of implementing such a system. We note that Siegler et al [30] documented that a substantial proportion of US respondents indicated they would they would be willing to submit participant-collected diagnostic specimens but were less willing to go to a drive-through, laboratory, or clinical setting to provide specimens. Therefore, the availability of at-home testing may increase our ability to test large numbers of people, including some who may be unwilling to go into clinical settings where they perceive themselves to be at risk for acquiring SARS-CoV-2 infection. Other countries have similar laboratory capacities, and some already use mailout specimens in public health programs: Public Health England uses mailout specimen collection and specimens returned by mail to screen asymptomatic people for sexually transmitted infections [37]. The ability to test people who have no or mild symptoms or are not willing to be tested in clinical settings can also reduce bias in estimates of SARS-CoV-2 prevalence that are generated from testing cohorts that are largely selected for symptomatic disease or the severity of that disease. Finally, there is a substantial benefit to developing and deploying testing methods for SARS-CoV-2 that are not reliant on supplies of rigid swabs, viral transport media, or PPE, all of which have substantial supply chain limitations. The self-collection of specimens at home thus limits the risk of exposure to health care providers, limits the extent to which PPE is used for diagnostic rather than care purposes, and reduces the congregation of people presenting for SARS-CoV-2 testing in clinics, where they run the risk of being exposed to other infectious patients.

### Limitations

Our study has important limitations. Our participants represent a biased group relative to the US population because they were included in the study based on their willingness to self-collect and return specimens. However, most of the 1435 respondents to an online survey reported willingness to collect and submit these specimens [30]; therefore, the extent of this bias may be minimal. We also acknowledge that the behavior of participants when collecting their specimens may have been influenced by the fact that they were being observed by a clinician (eg, a Hawthorne effect [38]). There are potential concerns about the shipping of boxes handled by participants who may be infected with SARS-CoV-2; however, the Centers for Disease Control and Prevention (CDC), the World Health Organization, and the US Surgeon General have indicated that there is no evidence for the spread of the virus that causes COVID-19 through the mail [32]. Our conclusion is that the specimens collected by the participants contained sufficient biological materials to support testing for RNA and antibodies; however, we do not report the results of our testing for SARS-CoV-2 RNA or serology. The CDC considers OPS to be a suitable specimen type if a nasopharyngeal swab is not available [33], and the FDA has granted an EUA for the use of saliva specimens [32].

There are important next steps to realize the promise of participant-collected specimens as one part of a suite of testing options available to address the current global pandemic of SARS-CoV-2. As noted above, it is important to conduct stability testing and to characterize the safety of the kit components before they are sent out to be used for self-collection without clinician observation. There is also a need for further studies to characterize the performance of serology testing for antibodies to SARS-CoV-2, and there are gaps in knowledge about the interpretation of those results. For example, we do not yet know the extent to which antibody responses confer partial, full, or no protection against reinfection. However, the possibility of new mechanisms to collect large numbers of samples from populations in difficult-to-reach places (eg, rural areas, during stay-at-home guidance) and from patients who are not symptomatic could have a practical public health impact. Potential applications of this technology include enabling the collection of specimens from large probability samples, monitoring the antibody status of communities through community sampling, establishing data on antibody kinetics by collecting serial (eg, daily) DBS collections mailed in by people who have been diagnosed with SARS-CoV-2 infection, and conducting screening of populations where it may be impractical to perform frequent health care visits.

### Conclusion

We collected and evaluated specimens that were collected by participants observed by clinical observers that can be used for diagnostic testing related to SARS-CoV-2 infection. Our data indicate that participants were willing to collect specimens and that clinical observers believe that the specimens collected only with reference to the provided instructions were suitable for laboratory testing. We believe that these data are generalizable to any participants who need to be tested for SARS-CoV-2 who have access to mail. Additionally, the laboratory assessment indicated that the DBS specimens were sufficient for testing and that the total nucleic acid content of the saliva samples and pharyngeal swabs were sufficient for testing and were consistent with the amounts of nucleic acid in physician-collected pharyngeal swabs and physician-observed saliva specimens. We believe that the potential benefits of the broad availability of participant-collected and mailed-in specimens for clinical purposes and for epidemiological monitoring of the COVID-19 epidemic in the United States outweigh the concerns about whether clinician-collected or clinician-observed at-home specimen collection will produce superior samples. One important issue from a workforce standpoint is defining the level of health care professional who should be recommended to observe self-specimen collection if telehealth-observed self-collection is implemented as a specimen collection method. Based on our observations of the specimen collection behaviors, and bearing in mind that clinicians did not intervene to correct participants who made mistakes, we believe that a broad range of medical professionals, including medical assistants, would be well prepared to fill this role. A final recommendation is to consider feedback from the test kit users; we collected this feedback but did not summarize it as part of this report. Further studies are needed to establish the safety and stability of the specimens during shipment. If procedures can be created that demonstrate safety and stability, we urge consideration of FDA review and approval of the use of participant-collected mail-in specimens for SARS-CoV-2–related diagnostics.

## Abbreviations

CDC: Centers for Disease Control and Prevention
COVID-19: coronavirus disease
C_t_: cycle threshold
DBS: dried blood spot
EUA: emergency use authorization
FDA: Food and Drug Administration
OPS: oropharyngeal swab
PPE: personal protective equipment
PrEP@Home: Pre-Exposure Prophylaxis at Home
RNase P: ribonuclease P
RT-PCR: reverse transcriptase–polymerase chain reaction
SARS-CoV-2: severe acute respiratory syndrome coronavirus 2
